# A proposed retinal venular immune gate: a mini review of perivascular macrophages and basement-membrane organization

**DOI:** 10.3389/fimmu.2026.1970491

**Published:** 2026-10-12

**Authors:** Zhiqing Huang, Zijian Huang

**Affiliations:** 1 Department of Ophthalmology, The First Hospital of Putian City, Putian, China; 2 Clinical College, North Henan Medical University, Xinxiang, China

**Keywords:** basement membrane, blood-retinal barrier, immune gate, leukocyte trafficking, microglia, perivascular macrophage, postcapillary venule, retinal inflammation

## Abstract

Leukocyte entry into the retina involves more than increased vascular permeability. This mini review synthesizes predominantly murine experimental evidence for a proposed retinal venular immune gate; the model has not been validated in the human retina. Recent studies place putative resident perivascular macrophages (pvMacs) near superficial postcapillary venules and basement-membrane low-expression regions (LERs), where matrix staining and pericyte coverage are reduced. These observations suggest that local immune signaling could interact with vascular architecture to concentrate leukocyte passage. Functional findings, however, diverge. Macrophage depletion reduces monocyte recruitment after an imposed intravitreal CCL2 challenge, whereas endotoxin-induced recruitment persists after depletion. The difference permits both stimulus-dependent macrophage participation and an alternative in which endothelial activation and permissive matrix regions govern entry without an essential macrophage organizer. We examine chemokine signaling, matrix binding, and antigen presentation as possible links, distinguishing retinal findings from mechanisms established in other tissues. In autoimmune uveitis, selective microglial MHC II studies identify an amplifying function while leaving the cellular control of earliest ocular entry unresolved. Human translation requires vessel-linked cell identification because clinical macrophage-like signals do not distinguish pvMacs from other myeloid populations. Comparing trafficking, permeability, and vascular repair after selective perturbation could reveal when perivascular macrophages influence entry and when they chiefly respond to it.

## 1 Introduction

Inflammation is often described as breakdown of the blood-retinal barrier (BRB). Yet the movement of water and plasma proteins differs from the passage of a living cell. A leukocyte must arrest, cross endothelium, negotiate basement membrane, and enter tissue. Asking where these events occur can therefore reveal biology that measurements of leakage alone leave unresolved.

Sterling and colleagues mapped retinal perivascular macrophages to superficial postcapillary venules and found reduced CCL2-induced monocyte infiltration after Pf4-based depletion (1). Lemire and colleagues subsequently associated these cells with venular basement-membrane low-expression regions (LERs). Leukocytes crossed at these sites during endotoxin-induced uveitis (EIU), but depletion did not reduce recruitment (2). Together, the studies invite a spatial account of retinal inflammation while raising a causal question: do macrophages organize passage, facilitate selected responses, or occupy sites made permissive by the vessel wall?

We examine a proposed retinal venular immune gate as a working model of interactions among endothelium, basement membrane, pericytes, and adjacent macrophages. Its value is the prediction that the arrangement of these components matters, not simply their abundance. The review develops that prediction alongside explanations in which macrophages are dispensable. **Figure 1** outlines the concept and the contrasting experimental findings.

**Figure 1:**
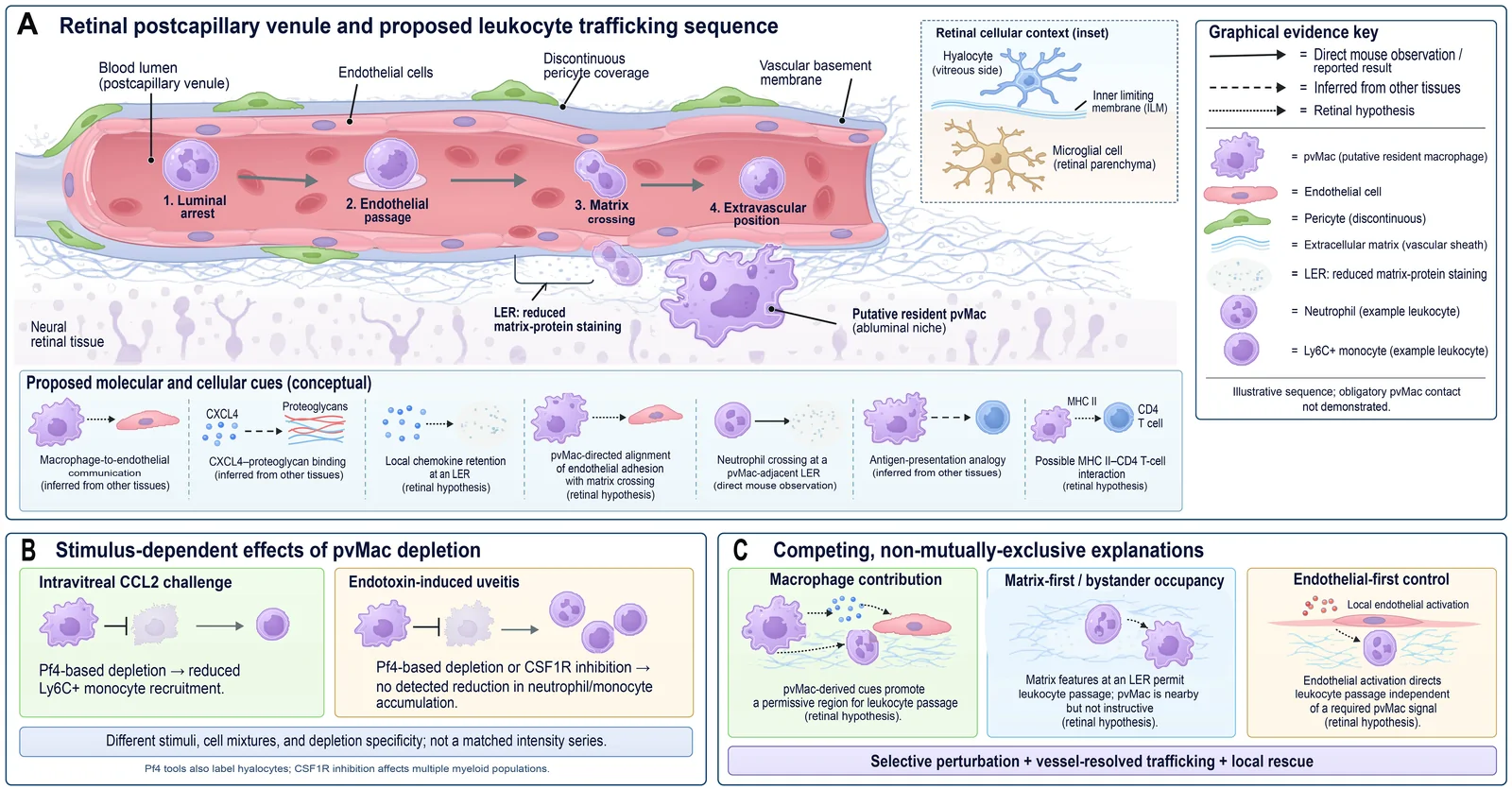
Proposed retinal venular immune gate and competing mechanisms of leukocyte trafficking. **(A)** Spatial model of leukocyte passage across a superficial retinal postcapillary venule, progressing from luminal arrest and endothelial transmigration to basement-membrane crossing, an extravascular position, and entry into neural retinal tissue. Putative resident perivascular macrophages are positioned in the abluminal venular niche adjacent to basement-membrane low-expression regions, which represent areas of reduced matrix-protein staining rather than fixed anatomical openings. The inset distinguishes pvMacs from hyalocytes at the vitreoretinal interface and microglia within the retinal parenchyma. Proposed molecular interactions include macrophage–endothelial communication, CXCL4 binding to matrix proteoglycans and local chemokine retention, alignment of endothelial adhesion with permissive matrix regions, neutrophil passage at pvMac-adjacent LERs, and a potential MHC II–CD4 T-cell interaction. **(B)** Contrasting effects of macrophage depletion under different inflammatory stimuli. Pf4-based depletion reduces Ly6C-positive monocyte recruitment after intravitreal CCL2 challenge, whereas Pf4-based depletion or CSF1R inhibition produces no detected reduction in neutrophil or monocyte accumulation during endotoxin-induced uveitis. These experiments differ in inflammatory stimulus, recruited-cell composition, and depletion specificity and therefore do not constitute a matched intensity series; Pf4-based approaches also affect hyalocytes, whereas CSF1R inhibition affects multiple myeloid populations. **(C)** Three non-mutually exclusive explanations for leukocyte entry: a macrophage-contribution model, in which pvMac-derived signals promote a locally permissive site; a matrix-first/bystander model, in which LER architecture permits passage while adjacent pvMacs are not instructive; and an endothelial-first model, in which local endothelial activation determines passage without an obligatory pvMac signal. Selective perturbation combined with vessel-resolved trafficking measurements and local rescue could distinguish these mechanisms. Solid connectors indicate direct mouse observations or reported experimental results, dashed connectors indicate mechanisms inferred from other tissues, and dotted connectors indicate retinal hypotheses. CCL2, C-C motif chemokine ligand 2; CD4, cluster of differentiation 4; CSF1R, colony-stimulating factor 1 receptor; CXCL4, C-X-C motif chemokine ligand 4; EIU, endotoxin-induced uveitis; ILM, inner limiting membrane; LER, low-expression region; Ly6C, lymphocyte antigen 6 complex locus C; MHC II, major histocompatibility complex class II; Pf4, platelet factor 4; pvMac, perivascular macrophage.

### 1.1 Literature identification

This narrative review used targeted web searches of PubMed-indexed records, PubMed Central, and primary publisher sites through 30 June 2026, without a lower publication-date limit. Terms combined “retina” or “retinal” with “perivascular macrophage”, “venule”, “basement membrane”, “low-expression region”, “CCL2”, “uveitis”, “MHC II”, “Tmem119”, “P2ry12”, and the disease names discussed below. Searches and reference checking prioritized primary spatial-imaging, lineage, selective-perturbation, depletion, and human-imaging studies, including discordant findings. Selection was narrative rather than systematic; no exhaustive screening or pooled analysis was undertaken.

## 2 Identity, anatomical position, and ontogeny

Here, “putative resident retinal perivascular macrophages” (pvMacs) denotes the macrophage population present along superficial venules before experimental inflammation. “Pre-existing” describes that observation without assigning embryonic origin or lifelong self-maintenance. Earlier work described autofluorescent perivascular scavenger cells in mouse and human retina (3). Their phenotype differed from modern reporter-defined pvMacs, so historical descriptions and contemporary populations should not be merged solely because both lie near vessels.

In mice, pvMac identification combines an abluminal venular position with CD64 and IBA1 expression, absent TMEM119, dim-to-intermediate CD206, and low or absent CD169. Pf4-based reporters label many pvMacs but also hyalocytes and a small microglial fraction (1). These shared labels matter when interpreting depletion. Surface location is equally important: vitreoretinal macrophage-like signals can include several myeloid populations, particularly during inflammation (4). Human retinal microglia have a molecularly characterized identity, whereas an equivalent vessel-linked human pvMac signature remains to be established (5).

Hyalocytes occupy the vitreal side of the inner limiting membrane, microglia the neural parenchyma, and choroidal macrophages a separate compartment outside the inner retinal circulation. Mouse fate mapping and parabiosis identify hyalocytes as yolk sac-derived, self-maintaining cells (6). Work on central nervous system border macrophages likewise demonstrates niche-dependent postnatal specification (7). These findings offer approaches for resolving retinal pvMac origin, rather than a lineage assignment by analogy. **Table 1** separates anatomy, mouse markers, and species-specific evidence. Recruited monocyte-derived perivascular macrophages are named explicitly throughout, because acquisition of a perivascular position does not establish membership of the pre-existing pvMac population.

**Table 1:** Ocular myeloid populations relevant to the proposed venular model.

Population	Anatomical niche	Working features in mice	Species/evidence source	Interpretation
Retinal microglia	Neural parenchyma; below the inner limiting membrane	TMEM119+, P2RY12+, CX3CR1-high at baseline; phenotype changes with activation	Mouse localization (1, 4); human retinal molecular profiling (5)	Perivascular proximity during inflammation does not by itself establish pvMac identity.
Putative resident retinal pvMacs	Abluminal sheath of superficial postcapillary venules before inflammation	CD64+, IBA1+, TMEM119−; CD206-dim/intermediate; CD169-low/negative; often Pf4-reporter+; MHC II expression	Mouse reporter, protein imaging, and spatial studies (1, 2); no corresponding validated human vessel-linked signature	“Pre-existing” describes baseline presence, not proven ontogeny. Pf4 tools also target other cells.
Hyalocytes	Vitreal side of the inner limiting membrane	TMEM119−, CD206-high, CD169+; frequently Pf4-reporter+	Mouse localization and fate mapping/parabiosis (1, 6)	Mouse evidence supports yolk sac origin and self-maintenance. A major confounder for Pf4-based experiments and surface imaging.
Recruited monocytes and monocyte-derived macrophages	Blood, neural tissue, or a secondary perivascular position during disease	Ly6C and CCR2 identify inflammatory subsets; later phenotypes vary. Some reparative perivascular cells accumulate independently of CCR2.	Mouse recruitment and disease studies (4, 19, 21)	Name recruited monocyte-derived perivascular macrophages explicitly; do not equate them with baseline pvMacs.
Choroidal macrophages	Choroidal stroma, outside the inner retinal vascular compartment	IBA1+; heterogeneous CD206 and other myeloid-marker expression	Mouse ocular myeloid and CSF1R-depletion studies (15)	Anatomically separate population; susceptibility to CSF1R inhibition complicates attribution of depletion effects.

Marker combinations are working definitions, principally from mice, and may change with inflammation. Species labels refer to the evidence supporting each description. pvMac, perivascular macrophage; MHC II, major histocompatibility complex class II.

## 3 Venular basement membrane and leukocyte passage

Basement membrane is a heterogeneous barrier. In mouse cremasteric venules, neutrophils preferentially traverse regions with reduced laminin, collagen IV, and nidogen staining, often aligned with gaps between pericytes (8). Monocytes and neutrophils share aspects of this route but differ in their interaction with and remodeling of the matrix (9). In retinal venules, LERs also show reduced matrix staining and frequently coincide with discontinuous pericyte coverage; three-dimensional imaging places extravasating neutrophils at pvMac-adjacent LERs (2).

LERs are permissive basement-membrane regions defined by relative matrix-protein immunostaining rather than by a demonstrated permanent anatomical opening. During EIU, retinal LERs enlarge and become less circular, and extravascular neutrophils carry collagen IV signal. Pf4-based depletion changes LER circularity without a detected change in area or collagen IV intensity (2). This combination suggests dynamic remodeling, with contributions potentially arising from the cells crossing the wall as well as those already beside it.

Endothelial passage remains a distinct control point. In a recent EIU study, platelet endothelial cell adhesion molecule (PECAM) blockade in BALB/c mice reduced neutrophil and T-cell transmigration; the investigators selected this strain because PECAM dependence differs between mouse strains (10). Such results make endothelial mechanisms a substantive alternative to macrophage-led organization. An informative spatial analysis would measure luminal adhesion, completed transmigration, and matrix crossing separately. Vascular permeability should be recorded in parallel, rather than used as a substitute for any of these events.

## 4 Potential macrophage contributions and stimulus-dependent recruitment

### 4.1 Chemokine signaling and contrasting depletion results

Pf4-expressing macrophage clusters analyzed by Sterling and colleagues contained chemotactic programs, including Ccl2 and other monocyte- and neutrophil-associated chemokines. The clusters suggest candidate mediators, although they do not establish secretion by each vessel-associated cell. After intravitreal CCL2 administration, infiltrating Ly6C-positive monocytes were spatially associated with pvMacs, and Pf4-based depletion reduced infiltration (1). This is a functional association between a targeted macrophage compartment and recruitment, with cellular specificity constrained by the accompanying labeling of hyalocytes.

The CCL2 experiment imposed an acute chemokine stimulus: 1 ng was delivered intravitreally and recruitment assessed 18 hours later (1). This intervention probes recruitment under an imposed chemokine gradient rather than the evolving endogenous signals of disease. EIU engages different inflammatory signals and recruits a different mixture of cells. In that setting, neither Pf4-based depletion nor broader colony-stimulating factor 1 receptor (CSF1R) inhibition produced a detected reduction in neutrophil or monocyte accumulation (2). These are not matched experiments along a single low-to-high inflammatory scale. Stimulus, cellular composition, depletion breadth, and observation time all affect their comparison.

One interpretation is that pvMacs contribute to selected chemokine responses while other signals sustain recruitment in EIU. Another is that permissive matrix regions and activated endothelium are sufficient for passage, with pvMac proximity reflecting niche preference rather than organization. Persistence of recruitment after depletion weighs against an obligatory macrophage-centered gate. It also makes the stimulus-specific version testable: selective interference with pvMac signaling should alter recruitment in a reproducible subset of conditions, not merely coincide with changes in total retinal inflammation.

### 4.2 Communication with endothelium

In inflamed intestinal mucosa, macrophage-derived TNF and endothelial TNF-receptor signaling organize neutrophil recruitment (11). This provides a mechanism by which an abluminal macrophage can influence luminal events. Retinal pvMac position and chemotactic programs make similar communication plausible, but the signaling direction has not been resolved in retinal venules (1). A local cytokine signal could align endothelial adhesion with a nearby permissive matrix region. Conversely, endothelial activation could recruit both circulating leukocytes and macrophage processes to the same site. Measuring which response precedes the other would distinguish these possibilities.

### 4.3 Chemokine retention within matrix

Biochemical and extra-retinal experiments show that CXCL4/PF4 binds extracellular-matrix proteoglycans and can promote recruitment without a conventional chemokine receptor (12). Retinal Pf4 expression supplies a possible connection between macrophage phenotype and local chemokine retention (1). The resulting hypothesis is that matrix binding concentrates a recruitment signal near a permissive region. A decisive measurement is whether LER-associated matrix retains more chemokine than neighboring regions. Mapping basement-membrane components, binding capacity, and bound chemokine abundance together could resolve this relationship. Retention could depend on the composition of the remaining matrix rather than on its overall density.

### 4.4 Antigen presentation and local amplification

Retinal pvMacs express major histocompatibility complex class II (MHC II), which identifies a potential route for interaction with CD4-positive T cells (1). In the brain, experiments involving microglia and perivascular macrophages implicate antigen presentation in CD8-positive T-cell recruitment (13). That MHC I-associated setting is distinct from a proposed retinal MHC II–CD4 interaction. For retinal pvMacs, antigen uptake, cognate presentation, and a resulting change in T-cell entry or retention remain separate questions. Local restimulation after crossing could amplify tissue inflammation without determining the first leukocyte crossing event.

## 5 Predictions of the model across disease contexts

### 5.1 Autoimmune uveitis

Experimental autoimmune uveitis (EAU) illustrates why initiation and amplification must be separated. Okunuki and colleagues found profound suppression of EAU after CSF1R-antagonist treatment and implicated retinal microglia in disease initiation (14). CSF1R antagonism, however, is not microglia-specific. Rajesh and colleagues documented depletion of choroidal macrophages with a CSF1R inhibitor in their ocular studies, including Supplementary Figure S2 in (15); Lemire and colleagues demonstrated depletion of both retinal pvMacs and microglia in their Figure 8 (2). These observations establish a specificity problem across ocular models, rather than proving that every inhibitor regimen affects every compartment identically. The EAU depletion phenotype cannot therefore be assigned exclusively to microglia.

The later study from the Connor/Ksander group used P2ry12- and Tmem119-driven conditional deletion of microglial MHC II. It reduced EAU severity and supported local restimulation of dendritic-cell-primed CD4-positive T cells (16). Conditional deletion attenuated the amplification phase rather than preventing initial disease, supporting microglial MHC II as an amplifying mechanism within the eye. Its less complete suppression than broad CSF1R antagonism is biologically informative, but the interventions remove different functions and cell populations. That contrast cannot identify the missing initiating cell, nor exclude MHC II-independent contributions from microglia.

Peripheral T-cell priming, the earliest ocular entry, and subsequent antigen-dependent expansion are thus distinguishable events. Earlier EAU studies localized adhesion-molecule changes and leukocyte recruitment to retinal vessels (17). The pvMac model predicts an additional local contribution to chemokine presentation or T-cell restimulation. Early endothelial signals and other local antigen-presenting cells offer competing explanations for initial entry. Selective pvMac perturbation coupled to early vessel-resolved imaging could test whether initial entry changes, whether later perivascular T-cell retention changes, or whether neither does. These outcomes would distinguish recruitment control from inflammatory amplification.

### 5.2 Diabetic retinopathy

CCL2-dependent monocyte trafficking contributes to BRB alteration in experimental diabetes (18), and CCR2-positive monocytes promote early retinal vascular lesions (19). These findings motivate examination of venular recruitment sites without establishing a pvMac–LER unit in diabetes. The spatial prediction is that selected venular segments acquire chemotactic activity and altered matrix organization before sustained cellular accumulation. Chronic vascular remodeling could also redistribute recruitment independently of pvMacs.

Human imaging reports increased macrophage-like cells in vision-threatening diabetic retinopathy and an association with macular edema (20). Such observations connect myeloid activity with clinically important disease, but the imaged cells cannot be assigned uniquely to pvMacs. Linking repeated images to vessel type, axial position, and subsequent infiltration would help determine whether the signal reflects venular recruitment, hyalocyte activation, or movement of microglia and recruited macrophages.

### 5.3 Retinal vein occlusion

In experimental retinal vein occlusion, recruited monocyte-derived perivascular macrophages protect endothelial cells against apoptosis. Their accumulation was not eliminated by CCR2 deficiency, whereas broad circulating-monocyte depletion increased endothelial injury (21). These cells are distinct from the pre-existing pvMac population described in uninflamed retina. The result introduces a temporal dimension: cells arriving through an inflamed vessel may later support its survival. A prediction worth testing is that the same venular region can sequentially host recruitment and repair, with lineage and function changing even when the anatomical position is similar.

### 5.4 Infectious retinitis and treatment-associated inflammation

Human pathological studies of cytomegalovirus retinitis implicate retinal vascular endothelial infection (22). They provide a vascular context for examining immune access, not a demonstration of pvMac-mediated recruitment. Here, a useful model must accommodate both tissue injury and pathogen control. Experiments that reduce leukocyte entry should therefore measure pathogen burden as well as retinal damage; reduced inflammation alone may obscure impaired defense.

Ocular recombinant adeno-associated virus studies reveal another sequence. Langer and colleagues observed microglia-independent initiation of vector-associated inflammation, persistent T-cell accumulation, and reduced inflammation with sphingosine-1-phosphate receptor modulation (23). This establishes a lymphocyte-centered mechanism in that experimental setting rather than a pvMac-specific one. Comparing early endothelial activation with later macrophage redistribution could test whether the perivascular niche contributes to entry, persistence, or resolution after vector delivery.

## 6 Alternative explanations and falsifiable predictions

The macrophage-organizer hypothesis predicts that altering pvMac function changes where or how efficiently leukocytes cross, beyond changes explained by the vessel itself. The bystander hypothesis instead predicts that pvMacs preferentially occupy LERs but do not determine passage. A selective perturbation that leaves endothelial activation, circulating leukocyte availability, and matrix structure comparable would separate these accounts. Unchanged spatial crossing patterns despite effective perturbation would favor the bystander account for that setting; altered crossing followed by restoration with local rescue would favor a functional contribution.

A matrix-first explanation predicts that LERs develop independently and attract both pvMacs and emigrating leukocytes. Longitudinal observations of LER formation before macrophage arrival, and persistence of passage at macrophage-free LERs, would support it. An endothelial-first explanation predicts that adhesion and junctional activation determine crossing sites once matrix permissiveness is considered. The PECAM results make this alternative experimentally credible (10). Comparing occupied and unoccupied LERs within matched venules would help avoid mistaking shared responses to vessel type for macrophage control.

Human colocalization offers an important anatomical test but cannot alone resolve causality. Multiplex imaging should map candidate pvMacs, endothelial identity, matrix composition, and pericytes in the same venular segments. Failure to find a reproducible vessel-linked population would challenge translation of the mouse model. A consistent population would support its anatomical basis; functional studies would then determine whether its signaling changes trafficking. Each outcome would refine the relationship between anatomical organization and cellular control.

## 7 Experimental priorities and candidate interventions

The most informative studies would connect cell identity to events in time. Fate mapping should distinguish pre-existing pvMacs from recruited monocyte-derived cells. Vessel-resolved imaging should quantify arrest, successful endothelial and matrix crossing, and abluminal retention for neutrophils, monocytes, and lymphocytes separately. Matched CCL2 and EIU experiments should characterize depletion in all ocular myeloid compartments and record circulating cell counts. Analyses should treat vessels as nested within animals or human donors rather than as independent biological replicates.

Human translation can build on label-free adaptive-optics imaging of retinal macrophage-like cell distribution and dynamics (24). Combining this with depth-resolved vessel registration and donor-tissue molecular maps would connect accessible clinical signals with anatomically identified cells. Spatial transcriptomics can supply candidate interactions, while protein localization and perturbation determine whether those interactions operate at the proposed site. This approach preserves the spatial information lost during tissue dissociation.

CCL2–CCR2 blockade, interference with chemokine–matrix binding, and controlled changes in matrix organization are candidate experimental tests, not validated therapies for a venular gate. Systemic CCR2 blockade could reduce blood-monocyte availability rather than local passage, and matrix manipulation could change permeability or endothelial survival independently of recruitment. Local delivery, compartment-resolved measurements, and functional rescue would help identify the relevant mechanism. Experiments should pair trafficking and leakage with vascular survival, debris clearance, and, where applicable, pathogen control. Success would mean modifying a harmful process without concealing the loss of a useful one.

## 8 Discussion

The proposed retinal venular immune gate brings a spatial question into the study of inflammation: what determines the site at which a leukocyte completes its journey across the vessel wall? Mouse studies provide compelling associations among pvMacs, venules, and permissive basement-membrane regions, but contrasting depletion results leave open how much control belongs to macrophages (1, 2). Their contribution may be stimulus-dependent, shared with other cells, or secondary to endothelial and matrix responses.

For humans, the central challenge is to connect a specific molecular identity with a specific venular position. Current macrophage-like imaging signals do not make that connection (4, 20, 24). Comparing competing explanations in the same spatial and temporal frame would connect venular position with lineage and changing cellular functions. Whether macrophages direct entry, sustain local amplification, or support repair, resolving these roles could explain why similar vascular appearances lead to different inflammatory outcomes.
